# Albumin infusion for the critically ill – is it beneficial and, if so, why and how?

**DOI:** 10.1186/s13054-015-0862-4

**Published:** 2015-03-30

**Authors:** Undurti N Das

**Affiliations:** UND Life Sciences, 2020 S 360th St, # K-202, Federal Way, WA 98003 USA; Department of Medicine and Bio-Science Research Centre, GVP Hospital, Gayatri Vidya Parishad College of Engineering Campus, Visakhapatnam, 533 048 India

## Introduction

Human albumin is used for volume expansion and resuscitation and to correct hypoalbuminemia [1]. Hypoalbuminemia is frequent in sepsis and is a strong predictor of mortality and morbidity [2]. Fluid volume expansion and resuscitation of the critically ill with albumin has been recommended by the UK National Institute for Health and Care Excellence and the Surviving Sepsis Campaign (grade 2C), although the evidence for these recommendations is not strong [1,3-8] and use of albumin to correct or improve hypoalbuminemia remains controversial [7,8] despite the fact that albumin is safe to use for the critically ill [8].

Albumin mobilizes polyunsaturated fatty acids (PUFAs) from the liver and other tissues, and thus enhances the formation of cytoprotective bioactive lipids – lipoxins, resolvins and protectins – that, in turn, suppress production of proinflammatory prostaglandins, free radicals and cytokines. The beneficial actions of albumin thus depend on its ability to mobilize PUFAs and the formation of adequate amounts of lipoxins, resolvins and protectins. For those who have hepatic and tissue deficiency of PUFAs, albumin fails to mobilize PUFAs and formation of lipoxins, resolvins and protectins will be inadequate, which may explain failure of the beneficial actions of albumin in the critically ill.

## Metabolism of polyunsaturated fatty acids and formation of lipoxins, resolvins and protectins

**Figure 1:**
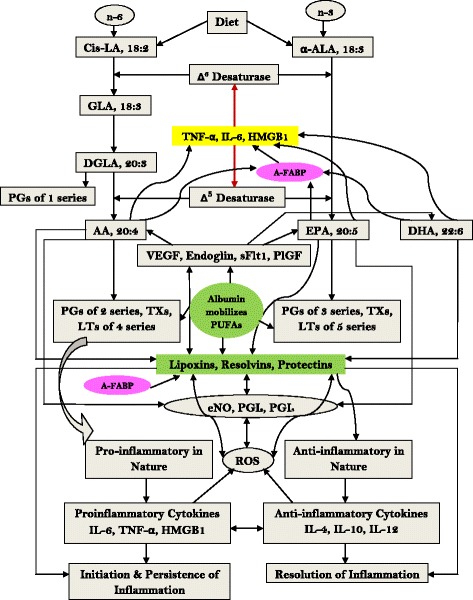
**Metabolism of essential fatty acids and their role in inflammation and its resolution.** AA, arachidonic acid; A-FABP, adipose-fatty acid binding protein; ALA, alpha-linolenic acid; DGLA, dihomo-gamma-linolenic acid; DHA, docosahexaenoic acid; eNO, endothelial nitric oxide; EPA, eicosapentaenoic acid; GLA, gamma-linolenic acid; HMGB1, high-mobility group box 1; IL, interleukin; LA, linolenic acid; LT, leukotriene; PG, prostaglandin; PlGF, placental growth factor; PGI, prostacyclin; PUFA, polyunsaturated fatty acid; ROS, reactive oxygen species; sFlt1, Soluble fms-like tyrosine kinase 1; TNFα, tumor necrosis factor alpha; TX, thromboxane; VEGF, vascular endothelial growth factor.

The cell membrane is rich in PUFAs (arachidonic acid, eicosapentaenoic acid and docosahexaenoic acid) that give rise to both proinflammatory prostaglandins, thromboxanes and leukotrienes and anti-inflammatory lipoxins, resolvins and protectins [9] (see Figure 1). Balance between these mutually antagonistic compounds could determine the final outcome of the disease process [9,10]. Lipoxins, resolvins and protectins reduce infiltration of leukocytes, suppress inflammation, show neuroprotective properties, inhibit oxidative stress-induced apoptosis of cells, inhibit proinflammatory cyclooxygenase-2 expression and tumor necrosis factor alpha (TNFα) production, enhance wound healing and promote brain cell survival by enhancing neuroprotective gene-expression programs [9-11]. Albumin enhances the formation of lipoxins, resolvins and protectins that are anti-inflammatory in nature and facilitate wound healing and resolution of the disease process [10,11] by mobilizing PUFAs. Hence, in albumin deficiency states decreased formation of lipoxins, resolvins and protectins occurs that may increase morbidity and mortality.

## Albumin has anti-inflammatory actions

Plasma concentrations of TNFα, interleukin (IL)-6 and macrophage inflammatory protein-2 were significantly lower and the IL-10 concentration higher in the albumin-treated hemorrhagic shock model animals [12]. Hypoalbuminemia thus results in decreased formation of IL-10 and lipoxins, resolvins and protectins that leads to increased morbidity and mortality [2]. The amount of PUFAs stored in the liver could therefore be one variable that influences formation of lipoxins, resolvins and protectins. Decreased activity of cyclooxygenases and 5-lipoxygenase, 12-lipoxygenase, and 15-lipoxygenase concerned with the formation of lipoxins, resolvins and protectins is yet another variability that contributes to the controversial responses to albumin therapy reported. The albumin half-life time is shorter and its transportation rate is higher in sepsis compared with control individuals [11], which results in inadequate availability of albumin to tissues in sepsis.

## Tumor necrosis factor alpha induces hypoalbuminemia and polyunsaturated fatty acid deficiency

TNFα administration to healthy well-nourished rabbits produced hypoalbuminemia [13] and endothelial cells treated with TNFα showed a decrease in their PUFA content and developed essential fatty acid deficiency [14]. These results suggest that enhanced circulating levels of TNFα and IL-6 seen in sepsis induce hypoalbuminemia and PUFA deficiency that leads to decreased production of lipoxins, resolvins and protectins. Furthermore, both vascular endothelial growth factor and placental growth factor that are increased in sepsis can be modulated by lipoxins, resolvins and protectins [15].

## Conclusions

Based on the preceding discussion, I propose that PUFAs need to be administered along with albumin to enhance formation of lipoxins, resolvins and protectins to derive the benefit of albumin therapy in the critically ill [16,17].

Beneficial actions of albumin are limited by: availability of PUFAs in the liver and other tissues; formation of adequate amounts of lipoxins, resolvins and protectins from PUFAs; activity of cyclooxygenase and lipoxygenase enzymes; circulating levels of TNFα and other cytokines that interfere with the formation of arachidonic acid, eicosapentaenoic acid and docosahexaenoic acid and induce hypoalbuminemia; enhanced formation of reactive oxygen species that peroxidize PUFAs and thus reduce their availability; and formation of proinflammatory prostaglandins that antagonize actions of lipoxins, resolvins and protectins [16,17] (see Figure 1). In view of the interactions among albumin, PUFAs, free radicals, prostaglandins, lipoxins, resolvins and protectins, nitric oxide (PUFAs and lipoxins enhance endothelial nitric oxide generation and protect endothelial cells), vascular endothelial growth factor, placental growth factor and cytokines, it is not surprising that albumin infusion alone is unlikely to be of benefit in sepsis. Hence, albumin needs to be given along with PUFAs and lipoxins to antagonize the actions of proinflammatory molecules and restore homeostasis [16,17].

Adipose-fatty acid binding protein (A-FABP), which induces insulin resistance and whose levels are increased in the critically ill [18], is a carrier of fatty acids and is expressed primarily in adipocytes and macrophages. A-FABP regulates systemic insulin sensitivity and lipid and glucose metabolism [19]. Mice deficient in A-FABP are resistant to the development of hyperinsulinemia, hyperglycemia and insulin resistance [18].

A-FABP can be linked to the expression of Toll-like receptors, macrophage activation, synthesis and release of proinflammatory cytokines IL-6 and TNFα, activation of cyclooxygenase-2 expression and eicosanoid synthesis, events that cause insulin resistance and initiation and progression of inflammation and sepsis. PUFAs and lipoxins, resolvins and protectins suppress A-FABP expression, inhibit macrophage and cyclooxygenase-2 activation, and decrease production of proinflammatory cytokines, and thus decrease insulin resistance and resolve inflammation and augment recovery from sepsis [18,19]. Hence, serial measurement of both proinflammatory and anti-inflammatory molecules and correlation of their levels with the progression to or recovery from (or both) sepsis and other inflammatory processes may help to predict prognosis in inflammatory conditions and eventually could lead to the development of new therapeutic strategies.

## Abbreviations

A-FABP: Adipose-fatty acid binding protein
IL: Interleukin
PUFA: Polyunsaturated fatty acid
TNFα: Tumor necrosis factor alpha
